# A Behavioral Paradigm to Evaluate Hippocampal Performance in Aged Rodents for Pharmacological and Genetic Target Validation

**DOI:** 10.1371/journal.pone.0062360

**Published:** 2013-05-07

**Authors:** Hilary Gerstein, Rikki Hullinger, Mary J. Lindstrom, Corinna Burger

**Affiliations:** 1 Department of Neurology, University of Wisconsin-Madison, Medical Sciences Center, Madison, Wisconsin, United States of America; 2 Neuroscience Training Program, University of Wisconsin-Madison, Madison, Wisconsin, United States of America; 3 Department of Biostatistics and Medical Informatics, University of Wisconsin-Madison, Madison, Wisconsin, United States of America; Pontifical Catholic University of Rio Grande, Brazil

## Abstract

Aged-related cognitive ability is highly variable, ranging from unimpaired to severe impairments. The Morris water maze (a reliable tool for assessing memory) has been used to distinguish aged rodents that are superior learners from those that are learning impaired. This task, however, is not practical for pre- and post-pharmacological treatment, as the memory of the task is long lasting. In contrast, the object location memory task, also a spatial learning paradigm, results in a less robust memory that decays quickly. We demonstrate for the first time how these two paradigms can be used together to assess hippocampal cognitive impairments before and after pharmacological or genetic manipulations in rodents. Rats were first segregated into superior learning and learning impaired groups using the object location memory task, and their performance was correlated with future outcome on this task and on the Morris water maze. This method provides a tool to evaluate the effect of treatments on cognitive impairment associated with aging and neurodegenerative disorders.

## Introduction

Aging is often associated with deficits in learning and memory. Rats, like humans, display a wide range of cognitive ability, ranging from learning unimpaired to severely learning impaired [1]–[5] and thus, can be used as a model for age-related cognitive impairments. The Morris Water Maze (MWM) spatial memory task can be used to segregate aged rats into superior learners (SL) and learning-impaired groups (AI) [1]–[8]. The MWM is a reliable test of spatial memory [9] and does not require food deprivation or electrical shock as a motivating factor. The major limitation of this method for separating aged rats by ability is that the memory for the MWM is long lasting and cannot be repeated in the same animals for at least four weeks, depending on the training regimen used, because of the possible carryover effects of previous training [10].

Research addressing the effects of memory enhancing drugs on the ability to learn hippocampal tasks has been carried out mainly using aged rats as a homogenous group that displays cognitive deficits relative to young animals. While this approach has been widely used, it is known that not all animals (or humans) show cognitive deficits with aging [6], [7], [11]–[15]. Labeling all aged rats as impaired has been used because there are currently no behavioral tasks that are forgotten quickly enough to be used to evaluate the level of individual impairment both pre- and post-treatment. In the case of the MWM, one alternative is to have two mazes in different rooms, or to wait over two-three months in between experiments, both of which can be impractical and costly [2], [10], [16].

One common test of spatial memory often used in rodents is the object location memory task (OLM). The OLM uses the natural preference of rodents for novelty to specifically assess spatial or location memory [17]–[21]. In this task, rats are exposed to two identical objects in two locations and then, 24 hours later, are exposed to the same two identical objects, one in the previous location and one in a novel location. Rodents usually will spend more time (∼60% of total) investigating the object in the novel location [22], [23]. Like the MWM, performance in the OLM task has been shown to be dependent on the hippocampus [17]. Unlike the MWM, the OLM task memory is less robust and can be relearned using new objects [18], [24], [25]. To our knowledge, it has not been shown that aged animals can be segregated into SL and AI groups using the OLM nor whether individual performance of SL and AI animals on this task is comparable to performance on the MWM.

We proposed to (1) use OLM to test spatial learning and classify naïve aged rats into SL and AI groups based on individual performance, (2) test whether OLM can be repeated and relearned shortly after the first round of OLM as a way to evaluate post-treatment effects on cognitive function, (3) test whether OLM can be used in conjunction with the MWM as a means to assess the effect of pharmacological or genetic manipulation on learning and memory using two independent hippocampal tasks [26]. Our behavioral method allows for the testing of markers for successful or unsuccessful cognitive aging. This will have a great impact on the field of Alzheimer’s disease (AD), where aged individuals with mild cognitive impairment (an intermediate stage between the expected cognitive decline of normal aging and the more pronounced decline of dementia), are believed to have a greater chance of developing AD than those without this impairment. The identification of markers that characterize these groups of aged impaired individuals will be facilitated by the use of this behavioral protocol.

## Methods

### Animals

Fifty aged (20-month old) and fifteen young (3-month old) F344 rats from the National Institute of Aging rodent colony were used in this study. All animals had free access to water and food. In addition, 12 hour dark and light cycles were maintained. Behavioral tests were given during the light cycle. All procedures were approved by the University of Wisconsin Institutional Animal Care and Use Committee and were conducted in accordance with the U.S. National Institutes of Health ‘Guide for the Care and Use of Laboratory Animals’.

### Object Location Memory

The experimental apparatus was made of clear Lucite, the outside of which was covered with dark blue construction paper, and measured 40.65 cm×40.65 cm×30.5 cm. Corncob bedding was spread ∼2 inches deep on the floor. To encourage exploration, direct overhead lighting was not used. Instead, knee-level fluorescent lighting was used to provide indirect illumination, and a standard desk lamp with a 60-watt incandescent bulb was angled at the ceiling over the experimental arena. On each day of the experiment, the arena and objects were cleaned with 70% ethanol and fresh bedding was put down to limit olfactory cues.

### OLM 1

On the first day (Habituation Day), all rats were habituated to the behavioral room and arena. There were no objects in the arena at this time and the rat was given 5 minutes to explore freely. The rat was then placed back in its home cage, any feces were removed, the bedding was mixed or stirred, smoothed down and habituation proceeded to the next animal. After all rats had been habituated for 5 min, the process was repeated in the same order. All rats received a total of two 5-min habituation exposures (with significant handling in the process) on this first day of the experiment.

**Figure 1:**
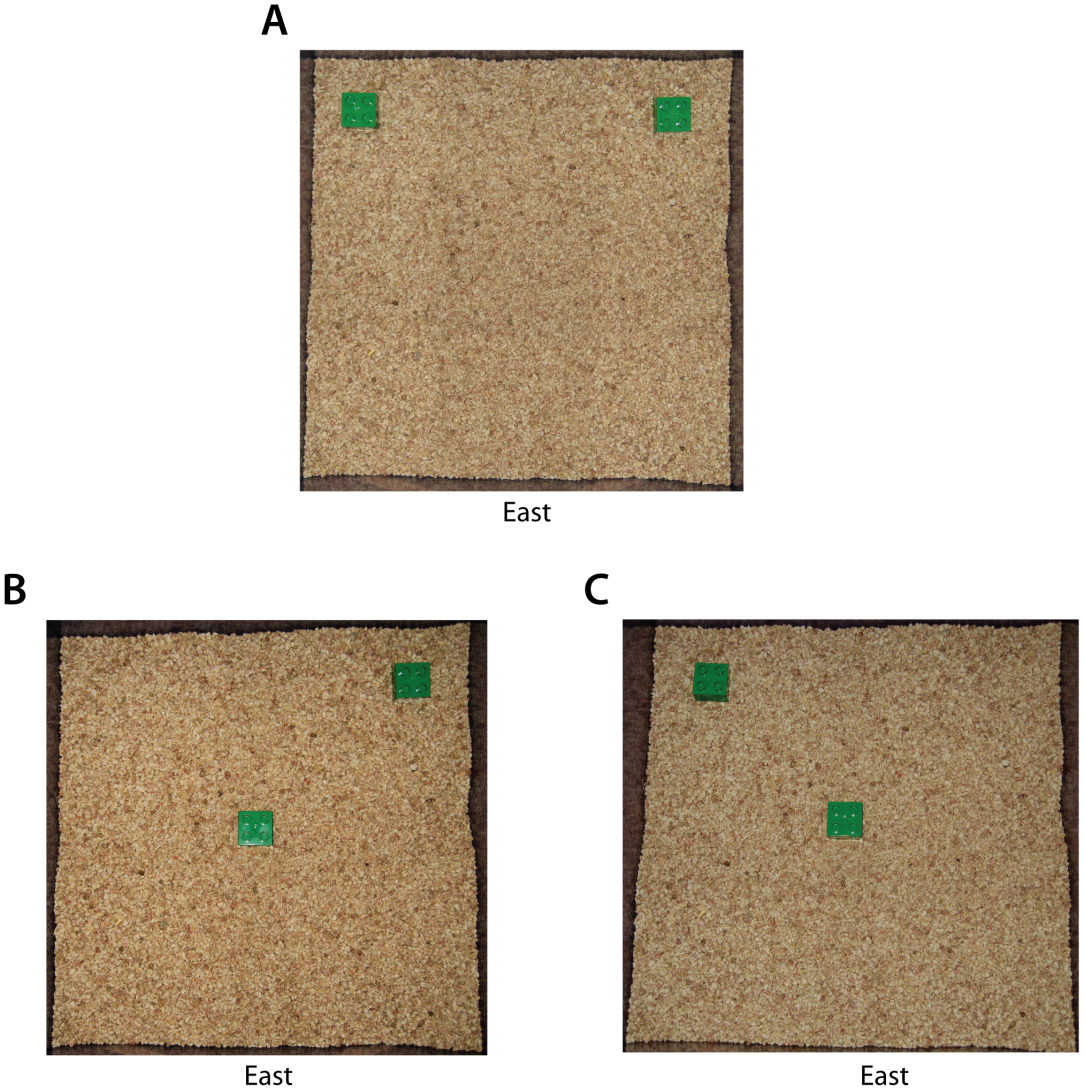
The OLM paradigm features novel and familiar object locations. Overhead view of OLM arena. (A) Object locations on Training Day. (B, C) Object locations on Testing Day.

On Training Day, 24 hours after Habituation, rats were trained on the locations of two identical objects. The arena was the same as previously described but with the addition of two identical objects (Duplo™ plastic blocks, 1.25×1.25×1 inch) in corner locations A and B (Fig. 1A), approximately 2.5 cm from the sides of the arena. The orientation of the box is such that the side featuring a spatial cue (X marked in tape) is East. Rats were allowed to explore the arena and the two objects freely over the course of a 10-minute trial. The bedding was stirred and the blocks were cleaned with 70% ethanol, before moving on to the next animal. Any animal failing to investigate both objects on the Training trial or whose total investigation time on the Training trial was less than 10 s, was excluded from the analysis so as to avoid confusing very low activity with low novelty-seeking behavior.

Testing of OLM occurred 24 hours after training. Spatial memory in the rats was tested by measuring their preference for the object in a novel location versus a familiar location. The arena was arranged as before with the same identical objects (Duplo™ blocks) and fresh bedding. However, in each trial, one of the two objects (A or B) was placed in the center of the arena instead of its original location (Fig. 1B). The location of the objects was counterbalanced, with half the rats presented with the ‘A in Center’ option, the other half presented with the ‘B in Center’ option. All combinations and locations of objects were used in a balanced manner (among experimental groups) so as to reduce potential biases due to preference for particular locations or objects. The orientation of the box was the same as on the previous day (Fig. 1B, C). The experiment was run similarly as during training, with 10-minute trials. It is important to comment on the selection of the novel location. Aged rats tend to have less locomotor activity than young rats [27]. For the new object location, we chose to place the object in the middle of the arena, which is close to one of the old locations, in order to encourage exploratory behavior while minimizing need for large amounts of locomotion.

All trials on both the Training and Testing days were videotaped and analyzed by an experimenter blind to the identity of the rat, using Videotrack software by ViewPoint Life Sciences (Montreal, CANADA). Total amount of time spent exploring the novel and familiar objects was recorded for each animal as described [18], [23], [25]. Briefly, a rat was scored as exploring an object when its head was oriented toward the object within a distance of approximately 1 cm or when the nose was touching the object. Sitting on, standing on, or sniffing the air above an object was not scored. The relative exploration time was recorded for each object and expressed as a novelty index: [(Time Spent (s) Investigating Object in Novel Location/Time Spent (s) Investigating Both Objects in Total) ×100]. Investigation times were calculated and novelty indices were examined in both young and aged animals.

In initial pilot studies utilizing young rats, no preference was seen for specific regions of the arena or for specific objects with our experimental setup. However, in the first experimental cohort run (including 15 aged animals) we found that the path of approach to the arena that the experimenter walked affected performance and exploration of the aged animals (and did not affect the young). The aged subjects preferred the opposite side of the arena from where the experimenter approached, thus also spending the majority of their investigation time exploring the object on that side of the arena (regardless of novelty). Moreover, for 13 of the 15 aged animals in this group, total investigation time did not meet the 10 s criteria and needed to be removed. Thus, OLM1 data from all aged animals in this cohort was not used in this study. Approach path to the arena and lighting conditions were optimized for OLM 2 and for the rest of the cohorts, so that rats in the arena could not see the experimenter until she appeared at the drop location in the center of the East wall, which improved exploration and removed any evidence of location or object bias in the aged animals. All subsequent instances of OLM (OLM1 in the second and third cohorts, OLM2 in all cohorts) were run in this manner and this is the data used for analysis in this study. 18 animals (15 from the first cohort, as explained above) had to be removed from the OLM1 analysis due to lack of overall investigation of objects, whereas only 4 were excluded from OLM2 for the same reasons. Therefore, OLM2 and MWM data are drawn from more aged animals than OLM1.

### OLM 2

All animals were allowed to rest undisturbed in the animal facility for 3 weeks before retesting in the OLM paradigm (OLM2). OLM2 and OLM1 were identical except that (1) during habituation the arena is placed in a different orientation (side with spatial cue is now West instead of East), (2) different objects were used (50 mL glass vials 1.5 inches in diameter, 2 inches tall spray painted light grey to enhance visual contrast with arena and bedding) and (3), each animal was presented with the opposite Testing Day position as on OLM1. For example, if a rat was presented with ‘A in Center’ on Testing Day during OLM1 (with Duplo™ blocks) then the same rat should be presented with ‘B in Center’ on Testing Day during OLM2 (with 50 mL glass vials) so as to counterbalance objects and locations within the arena.

### Criteria for Categorizing SL and AI in the OLM Task

Aged animals were identified as superior learners when their novelty index on the OLM task was at or above the mean score of young rats (59% was the young mean for OLM1, 63% was the young mean for OLM2). This resulted in choosing aged rats with the highest novelty indices (as they spent more time than other aged animals investigating the object in the novel location), whereas aged impaired learners were identified as animals that spent 51% (or less) of their total object investigation time attending to the novel object. For each individual, performance rankings in OLM2 were tabulated independently of performance in OLM1.

### Morris Water Maze

Morris Water Maze (MWM) testing began 2–3 days after conclusion of OLM2. The maze consisted of a dark blue tank, 173 cm in diameter. No dye was added to the water as all platforms used were made of clear Lucite. The water temperature was checked daily and kept at 20–22°C. Both clear Lucite hidden and visible platforms were 10 cm×10 cm, with the standing area submerged ∼5 cm below the surface of the water. During the first two days of water maze (Days 1–2), animals were acclimated to the task with a visible platform placed in the exact center of the pool. The visible platform trial consisted of four trials per day, each lasting 90 seconds. These training trials also served to test for visual acuity in aged rats, as this strain of rats (Fischer 344) are prone to retinal degeneration [28]. Additionally, any animals with visible cataracts were removed from the study (n = 1). The visual cue training was run with full view of the spatial cues used in the hidden platform training task. Following the two-day visible platform training, subjects were then trained in sets of four trials per day for eight days (32 trials total).

The hidden platform version of the MWM was performed on Days 3–10 of the task. On hidden platform trials, the platform was always located in the Southeast quadrant of the pool. For each trial, the rat was dropped into the tank facing the wall at one of four possible locations (North, South, East, West) and these drop locations were scrambled within each day, such that each drop location was used once on each day and the order of drop location was different each day. The length of the swim path to the escape platform (or distance traveled in cm) was analyzed and measured using Videotrack software by ViewPoint Life Sciences (Montreal, CANADA). If a rat was not able to find the platform after 90 seconds, he was guided to the platform and allowed to sit on it for 10 seconds before being removed and dried off. Animals remained on the platform for approximately 10 seconds before being removed and thoroughly dried with a towel. The rat was then immediately taken to the next drop location and the next trial began. At the end of all four trials for a subject on a given day, the rat was dried with a towel before being placed in a heated dry-off cage until thoroughly dry, then returned to its home cage. Animals were given four trials a day, except for the final day (Day 10) on which they were given four trials followed by a probe trial, as follows.

For the probe trial, the platform was removed immediately after the last hidden trial; the animal was reintroduced to the pool, and allowed to swim for 90 seconds. Percent of total distance covered (and time spent) in the target quadrant that previously contained the platform was measured. Number of platform crossings in the probe trial was calculated by tallying the number of times each subject entered the platform zone during the 90-second trial.

### Assignment to Categories in the MWM

Criteria for categorizing animals as SL and AI was based on probe trial performance: Animals were identified as aged superior learners when 40% (or greater) of total swim distance (cm) was spent in the target quadrant that previously contained the platform, which corresponded to the bottom of young performance range. Impaired learners spent approximately chance percentages of their total swim distance in the target quadrant [25% of individual’s total+SEM of young performance]. For each individual, performance ranking in the MWM probe trial was tabulated independently of performance in OLM1 and OLM2.

### Statistical Methods;General

Nonparametric rank based correlation analysis was used in all cases. McNemar’s test was used to test the hypothesis that two categorical variables are creating significantly different categorizations.

The continuous variables MWM percent distance in target quadrant and number of platform crossings outcome were transformed to the log scale when used as response variables in linear models analysis (ANOVA and mixed effects models) in order to obtain constant variability over the range of responses. Since platform crossings can be zero, the transformation log(x+1) was used. All calculations were done in the R statistical language (R Development Core Team; 2008).

### Statistical Methods; MWM Learning Curve and Probe Trial

A two way linear mixed effects model was used to assess the ability of groups defined using probe trial distance results to predict the outcome of the learning phase. Terms included were day (categorical), group and their interaction. Hypothesis testing was accomplished using a parametric bootstrap supplied with the lme4 software package for R. Post-hoc pairwise analysis was accomplished by repeating the analysis with two groups at a time. One-way ANOVA was used to assess the ability of the probe trial categories based on percent distance to predict platform crossings, Tukey HSD post-hoc tests were used.

## Results

### Aged Animals can be Categorized as Superior Learners, Learning Impaired, and Intermediate Learners using the Object Location Memory Task

**Figure 2:**
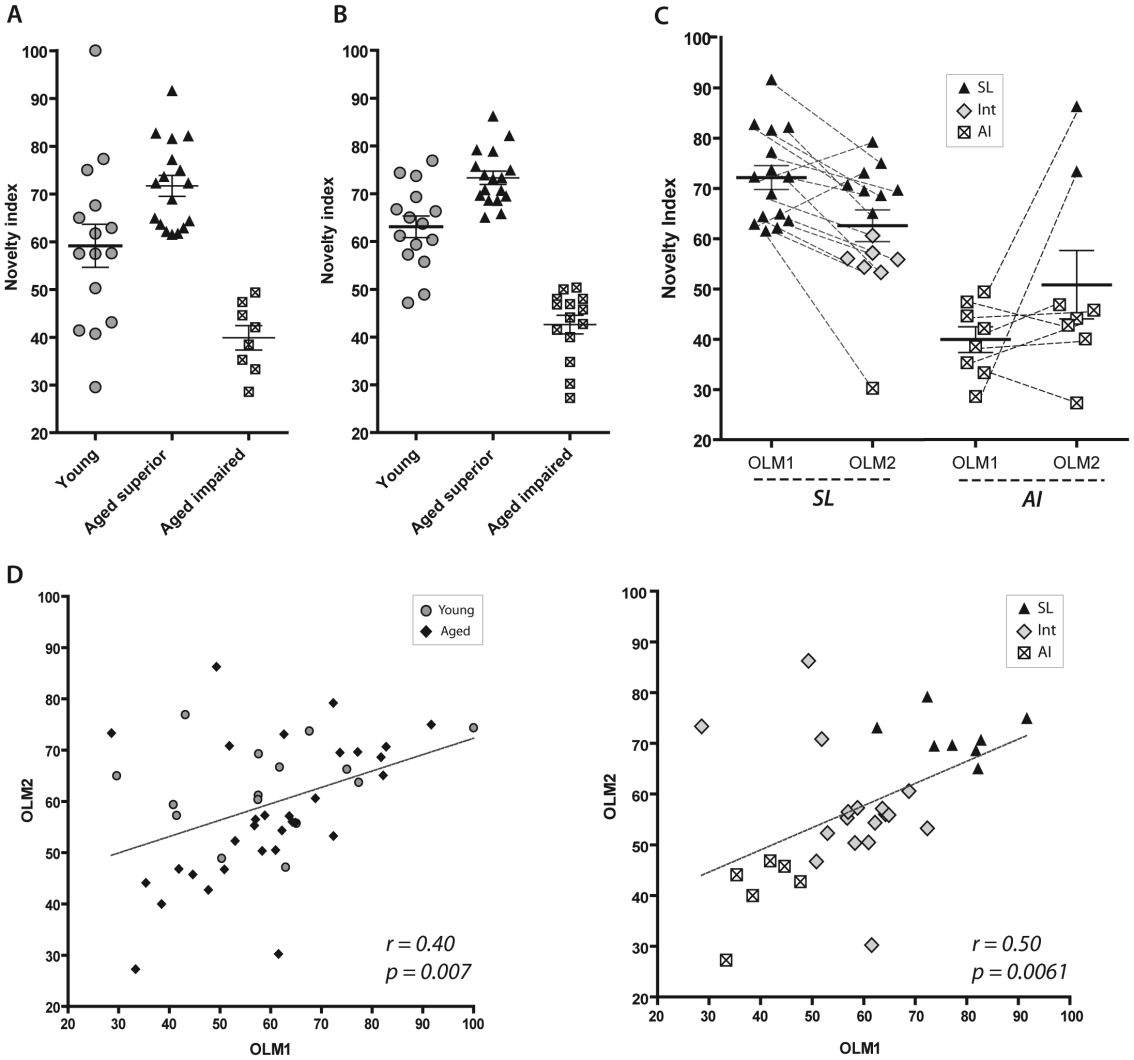
Statistical analysis shows a significant correlation between individual performance on OLM1 and OLM2. Data shown as novelty index, or proportion of total investigation time spent attending to the object in the novel location. (A) First round of OLM (OLM1) [young, n = 15; SL, n = 17; AI, n = 8]. (B) Second round of OLM (OLM2) using the same animals [young, n = 15; SL, n = 17; AI, n = 14]. Aged performance groups on OLM2 are delineated without reference to previous performance on OLM1. (C) For animals ranked as SL or AI on OLM1, a dotted line connects that individual’s performance on OLM1 to their performance on OLM2 (see also Table 1). Only animals classified as SL or AI on OLM1 are shown, and their classification on OLM2 are shown as SL = black triangles, AI = white squares, and intermediates = grey diamonds. (D) Correlation analysis between individual performance on OLM1 and OLM2. Young = grey circles, aged = black diamonds (n = 44, all young, SL, AI, and Intermediate animals included). (E): Correlation analysis for the aged rats. Grey diamonds indicate animals that were categorized differently in OLM1 and OLM2, or were classified as Intermediate in both tests. For details on this group of animals please refer to Tables 1 and 2.

Fifty aged (20-month old) and fifteen young (3-month old) F344 rats were used in this part of the study. Total amount of time spent exploring the novel and familiar objects was recorded for each animal and novelty indices were examined in both young and aged animals as described in detail in the methods section. Aged animals with a novelty index equal to or greater than the mean of the young animals (59%±4.5 S.E.M.) were labeled superior learners, those with a novelty index of 51% or less were classified as aged impaired learners, and those performing between the level of SL and AI were labeled as intermediate. For the first round of OLM, 53% of all aged animals (n = 32) were classified as superior learners, 25% as intermediate, and 22% as aged impaired (Fig. 2A).

Note that the classification of the aged rats requires a concurrently run group of young rats so as to define the cutoff value for SL (Fig. 2A).

When young animals were compared to the aged group as a whole using a student’s t-test, no significant differences were found (t = 0.2363, df = 45, p = 0.814). This shows that aged animals as a group are not significantly worse on this task relative to young animals.

### Performance on OLM1 is Correlated to Performance on OLM2

We investigated whether animals could relearn the OLM task with new objects three weeks later without exhibiting carryover effects from prior training. More importantly, we also wished to see if individual performance was maintained between first and second rounds of OLM (denoted as OLM1 and OLM2). For OLM2, we repeated the three-phase OLM1 task (habituation, training, and testing) for all animals using the same environment and arena but with novel sets of objects (painted 50 mL beakers). Novelty indices were calculated for all aged animals and were again separated into SL and AI groups, as described above, without consideration for their previous performance during OLM1. Aged animals showing a novelty index on OLM2 that was equal to or greater than the mean of young performance (63% ±2.2 S.E.M.) were classified as SL and 51% was again used as the cutoff for AI. On OLM2, 38% of all aged animals (n = 44) were categorized as SL, 29% as Intermediate, and 33% as AI. Thus, similar to OLM1, a proportion of aged rats are able to learn the OLM2 task successfully at a later time point with different objects (Fig. 2B).

When young animals were compared to the aged group as a whole in OLM2, no significant effect of age was found (t = 1.207, df = 57, p = 0.233).

Our data indicates that there is no carryover memory effect from prior training, as animals do not perform better as a group on OLM2 than in OLM1, and that they can relearn this task with new objects (Fig. 2A, B). Moreover, of the 15 aged rats classified as SL on OLM1, 8 of these animals were independently classified as SL again on OLM2. Of the 8 aged animals classified as AI on OLM1, 6 of these were again classified as AI on OLM2. Table 1 shows the cross tabulation of the category assignments for the two runs. McNemar’s test for paired data was not significant, providing no evidence that the classifications (SL, AI, and intermediate (INT)) are measuring different phenomena (p = 0.1789).

**Table 1:** Category assignments from OLM1 and OLM2 of the same group of aged rats shown as a cross tabulation.

	OLM2			
		*AI*	*INT*	*SL*
**OLM1**	***AI***	6	0	2
	***INT***	1	4	1
	***SL***	1	6	8

For example, 6 rats classified as AI by OLM1 were also classified as AI in OLM2.

Details on the fates of SL and AI animals, as classified from OLM1 and reassessed on OLM2, are shown in Figure 2C, with each individual’s performances connected by a dotted line. This graph shows consistency in individual performance on OLM1 and OLM2. We also assessed repeatability of the OLM task using correlation analysis on all young and aged animals (SL, AI, and intermediate groups). A significant correlation was found between individual performance on OLM1 and OLM2 performed 3 weeks apart (n = 44, r = 0.40, p = 0.0077) (Fig. 2D). Only animals that completed both rounds of OLM and were not excluded from either experiment (due to lack of exploration or premature death) were included in this analysis (young, n = 15; SL, n = 15; AI, n = 8, and intermediate, n = 6, as defined by OLM1) (see methods for details). Finally, we also investigated whether there was a correlation in performance when analyzing the three groups of aged rats (SL, INT and AI rats). When examining only these groups, we again found a significant correlation between performance on OLM1 and OLM2 (n = 29, r = 0.50, p = 0.0061) (Figure 2 E).

### Categorization of Aged Animals Based on Performance in the Morris Water Maze

**Figure 3:**
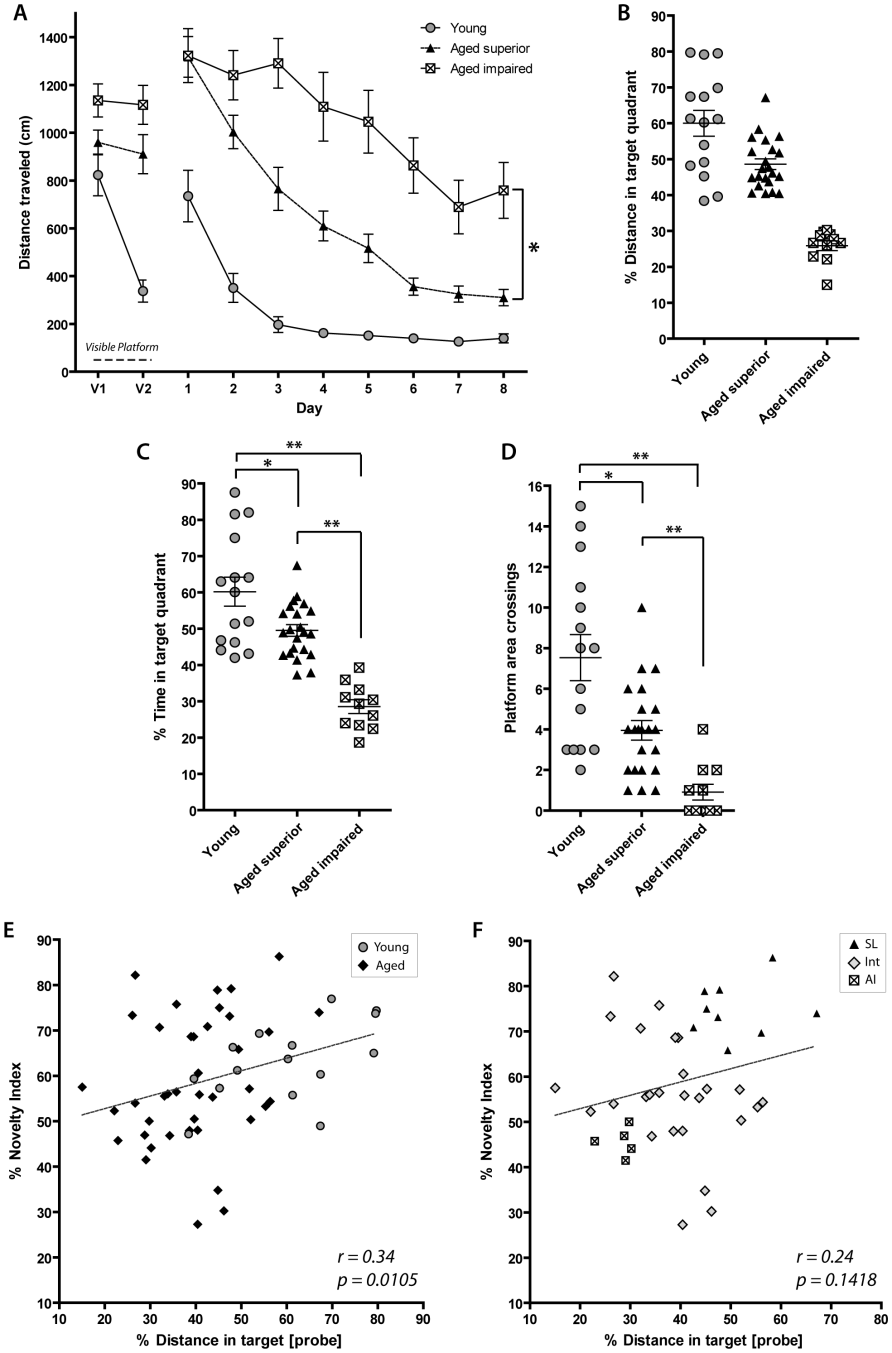
Performance on OLM correlates with performance on the MWM probe trial. (A) Task acquisition on the MWM in young and aged rats, four trials per day. The first eight trials (Days 1 & 2) represent the visual version of the task. Young animals learn the task quickly, although aged superior learners learn the task soon afterward. Aged impaired learners do not show high acquisition of the task. Asterisk indicates a significant difference of AI and SL performance on the MWM over time. (B) Percent of total swim distance spent in target quadrant during probe trial (Day 10) was the criterion by which animals were grouped into SL, AI, or intermediate. Both young and SL animals cover >40% of their total swim distance in the target quadrant. AI animals perform close to the chance level (25%). (C) Percent of total time spent in the target platform during probe trial show significant differences performance between SL, AI and young rats. (D) Platform crossings during probe trial show statistical differences in performance between SL, AI and young rats. (E) Individual novelty index on OLM2 significantly correlates to performance on the probe trial of the MWM when young (grey circles) and aged groups (black diamonds) are included (n = 55, young, SL, AI, and intermediate animals included). [Young, n = 15; SL, n = 22; AI, n = 11, and intermediate, n = 7, as defined by % total target swim distance]. (F): Correlation analysis for the aged rats. Grey diamonds indicate animals that were categorized differently in OLM2 and MWM, or were classified as intermediate on both. For details on these groups of animals, please refer to Tables 2 and 3.

The Morris water maze was performed in the same group of young and aged rats used for the two OLM tasks described above. Animals began the 10-day long MWM two days after completing OLM2. Days 1 and 2 consisted of habituation/training with a visible platform. The hidden platform version of the MWM was performed on Days 3–10 of the task, with four trials per day. Total swim path distance to find the escape platform on each trial was measured in centimeters and binned by day to assess task acquisition (learning curve) (Fig. 3A). At the end of the last training trial, the platform was removed and a probe trial was run in which the swim path of the rat was tracked for 90 s. Percent of total distance and time spent in the target quadrant (that previously contained the escape platform) and number of platform crossings (number of times the rat crossed the exact prior location of the platform) were measured in the probe trial (Fig. 3B–D). Aged rats were classified as SL, intermediate, and AI based on the percent of total distance spent in target quadrant during the probe trial. Animals were classified as SL when they spent 40% or more of their total swim distance in the target quadrant [the bottom of young performance range]. Animals were classified as AI when they spent approximately chance levels of total swim distance in the target quadrant [25% of total distance+S.E.M. from young performance] (Fig. 3B).

We found that SL aged rats improved markedly during the learning curve trials while the rats deemed to be AI demonstrated significantly impaired performance in the task, relative to the SL group. There was a significant effect of MWM group on distance traveled to the platform and that effect varied over the learning trials (significant group by day interaction MEM, parametric boot strap test, p<0.0001) (Fig. 3A). Post-hoc pairwise comparisons found the same result for each pair of groups (group by day interactions, parametric bootstrap test, p<0.0001).

MWM probe trial performance group (based on % distance in target quadrant) (Fig. 3B) is also a significant predictor of the percent time spent in the target quadrant and the number of platform crossings during the probe trial (one-way ANOVA, *F*_(2,45)_ = 29.5, p<0.0001, Fig. 3C; *F*_(2,45)_ = 26.8, p<0.0001, Fig. 3D; respectively). Post-hoc significant differences were found between all pairs of groups on both these measures as well (Tukey HSD, SL vs. AI and young vs. AI: p<0.0001, SL vs. Young: p<0.05, for both) (Fig. 3C, 3D).

When comparing young to old animals as a whole group in the probe trial of the MWM, we find a significant effect of age on performance (t = 5.616, dr = 56, p<0.0001) showing that aged animals, as a whole, perform more poorly in this task.

It is important to note that although aged rats did not perform as well as young rats in the visual version of the MWM, SL showed improvement in their ability to find the hidden platform over time and also showed spatial bias on the probe trial. Therefore, it is unlikely that visual or motor deficits caused the initial impairments seen in the aged animals on the visible platform trials. Rather this is likely to be an age-related slowness in task acquisition.

### Individual Performance in the OLM Correlates to MWM Performance

We assessed the correlation of individual performance in the OLM with performance in the more widely used MWM. Due to the high number of animals excluded from OLM1 (see methods) we used scores on OLM2 to correlate behavior on this task to individual performance on the MWM, as similar numbers of animals completed both of these tasks (45 and 43, respectively). We found a significant correlation between individual OLM2 novelty index and percent distance spent in target quadrant in the MWM probe trial when pooling all groups, young and aged (n = 55, r = 0.34, p = 0.0105) (Fig. 3E and Table 2). Significant correlation between individual OLM2 novelty index and platform crossings in the MWM was also seen (r^ = ^0.33, p = 0.0129; data not shown). Table 3 shows the cross tabulation for the category assignments for OLM2 and MWM. Correlation analysis using the three groups of aged rats (SL, INT and AI) shows no statistical significance (n = 40, r = 0.24, p = 0.1418) (Figure 2F).

**Table 2:** Raw data from OLM2 (novelty index) and MWM probe trial (% of total swim distance in target quadrant) illustrates the criteria used to categorize superior and impaired learning ability (one cohort shown of three).

OLM2				MWM Probe			
Rank	ID#	OLM Novelty Index	Group	Rank	ID#	% Distance	Group
**1**	13b	86.4		**1**	17a	67.1	
**2**	9a	82.6		**2**	13b	58.3	
**3**	11b	79.3		**3**	20a	49.4	
**4**	7a	79.0		**4**	11b	47.8	
**5**	10a	75.4	*Superior*	**5**	8b	47.4	*Superior*
**6**	17a	74.2		**6**	7a	44.7	
**7**	8b	73.2		**7**	7b	43.7	
**8**	16b	71.3		**8**	15b	40.6	
**9**	18a	68.5		**9**	19a	40.4	
**10**	20a	65.9		**10**	18b	39.7	
**11**	15b	60.6		**11**	18a	39.5	
**12**	10b	57.6		**12**	8a	38.6	
**13**	12b	56.4	*Intermediate*	**13**	10a	35.8	
**14**	14b	56.1		**14**	12b	35.7	*Intermediate*
**15**	14a	55.3		**15**	19b	34.3	
**16**	7b	55.3		**16**	14b	33.9	
**17**	6a	53.6		**17**	14a	33.1	
**18**	18b	50.5		**18**	16b	32.0	
**19**	12a	50.1		**19**	12a	29.8	
**20**	19a	48.1		**20**	13a	29.0	
**21**	8a	47.7	*Impaired*	**21**	20b	28.8	
**22**	19b	47.1		**22**	9a	26.7	*Impaired*
**23**	20b	46.7		**23**	6a	26.6	
**24**	9b	45.8		**24**	9b	22.9	
**25**	13a	41.8		**25**	10b	15.0	

Horizontal lines show cutoffs for superior, intermediate and impaired categories.

**ID#** - Rat identification number.

**Rank** - Animals have been ranked in order of performance from high to low.

**Table 3:** Category assignments from OLM2 and MWM (% distance spend in target quadrant in probe trial) of the same group of aged rats shown as a cross tabulation.

	MWM			
		*AI*	*INT*	*SL*
**OLM2**	***AI***	5	2	5
	***INT***	3	3	7
	***SL***	2	4	9

For example, 5 aged rats classified as AI by OLM were also classified as AI in the MWM probe trial.

## Discussion

In this study, we proposed to (1) investigate if the OLM task can be used to classify aged rats into groups based on performance, (2) show if this task can be reliably repeated in the same individuals (within a short inter-test interval), and (3) establish if performance on the OLM is consistent with performance on the MWM. Our results show that aged rats can be segregated into superior learning and aged impaired subpopulations using the OLM test of spatial memory and that individual performance on this task has a small but significant correlation with future OLM experiments, when using new objects. While the OLM paradigm has been used by several groups to discriminate performance between young and aged rodents [22], [24], [25], [29], to our knowledge this is the first report showing that this task can also be used to reliably segregate individual aged rats by ability. Moreover, we have demonstrated that performance in the OLM correlates with that on MWM, which is the commonly used paradigm for separating out AI and SL groups [9], [11], [15], [30], [31]. Thus, the OLM can be utilized to examine the effect of specific treatments on spatial memory, much like the MWM, but with the benefit of using a within-subjects behavioral design. In addition, the OLM task is not heavily dependent on motor function and thus removes the confounding effects of age-related decline in motor function in the assessment of memory.

It is worth noting that AI animals tend to spend less that the 50% random likelihood mark for memory investigating the object in the familiar location [32]. The fact that they do spend more time in the old location could be interpreted as thigmotactic behavior in the AI rats. We do not find this behavior in SL rats, as they spend a greater proportion of their time with the object in the novel location. We have shown previously that, when tested in the MWM, AI rats tend to be more thigmotactic that SL rats [7], [8]. Thus, part of the behavioral deficits in AI animals might include a tendency for thigmotaxis, or lack of pliancy to overcome this behavior with training [33]. However, since little or no thigmotaxis was seen in the SL animals (either in the current study or in prior studies utilizing superior learning aged rats [7], [8]) this is not a problem affecting aged rats as a group.

Interestingly, we found that there was no significant correlation between the OLM and MWM when the young rat group was excluded from the aged rat group analysis (SL, AI and intermediates; figure 3F). There is precedent for some aged animals performing better in the MWM than in the object recognition memory due to the heightened motivation to get out of the water in this task [27]. Indeed, five of the animals labeled AI in OLM2 were grouped as SL in MWM (Tables 2 and 3), although they were bottom SL performers in the MWM. This does not necessarily invalidate our approach, as using the MWM as a benchmark might result in an underrepresentation of AI animals. Therefore, the OLM task is a powerful test to identify this potential group of cognitively impaired animals that could be candidates for pharmacological or genetic treatment. In fact, using the OLM task to identify AI subjects, we showed that AI animals that received Homer1c gene replacement significantly improved performance in the OLM and in the MWM, relative to AI animals that received a control treatment [26].

We show that the OLM test can be repeated within a short time after OLM1 and that animals can relearn the task with new objects. For the first time, we show that this task can be used to segregate aged animals into SL and AI groups, just as others have shown with the MWM. This provides a good internal control for experiments where pre and post-drug treatment are performed. Tests like the MWM and fear conditioning produce long lasting memories and therefore are not optimal for performing within subject comparisons. Other alternatives for re-testing in the MWM include changing the MWM to a different room, changing the location of the platform, or testing the animals in the radial arm version of the MWM in subsequent testing [2], [10], [16], [34], [35]. However, the OLM test requires only two days of training, provides a reliable way to segregate animals into performance groups, and adds an additional test of spatial memory that can be used to assess the effects of treatments for recovery of cognitive function. The OLM task exploits the natural preference of animals for novel locations and can be used in conjunction with the MWM as an additional test for hippocampal learning.

Our study is unique in its comparison of individual performance on the OLM and MWM paradigms. Prior work has been performed in which the same individuals were used for different tests of spatial memory, such as the MWM and those involving food as a reward (T-maze or radial arm maze) [36], fear conditioning [37], inhibitory avoidance [14], operant conditioning [11] or radial arm maze [38]. However, the present study is the first to compare aged group performance in the OLM and the MWM and to find consistency in aged rat performance between these two different hippocampus-dependent behaviors. Our data, together with these abovementioned studies, are suggestive of a psychometric g factor for rodents where there are positive correlations or “concordance” between individual performance in different behavioral tasks [32], [39], [40]. Since both OLM and MWM test hippocampal function, it will be important to increase the number of tests of cognition to prove this general learning ability in aging rodents and that performance in the various behavioral tasks are affected by a general factor instead of a domain-specific (i.e. hippocampal function-related) factor [40]. This will be relevant in light of the fact that aging might affect hippocampal function more than function involving other brain regions [41].

In our study, young animals performed consistently well in the MWM, with a low degree of behavioral variability, as we and others have shown [1]–[8]. Variability in individual performance in the MWM increases with aging, so deficits in the MWM are not detected until late in life [5], [12], [40]. However, in this study we observed measurable difference in individual performances of young animals in both rounds of the OLM task (Figure 2). This suggests that young animals do show selective differences in spatial memory, but that currently used Morris water maze paradigms are not able to reveal these natural differences in young healthy rodents. It will be interesting to investigate whether young animals that show lower ability early in life in OLM will be more likely show impairments in this and other memory tasks with aging. As the OLM task can be used repeatedly by changing objects, it should be straightforward to test these animals at several points during their lifespan to answer this question.

In conclusion, we have demonstrated that the OLM task can be repeated to assess individual performance in the same group of rats, and that performance on OLM correlates with performance on the MWM when young and aged groups are compared; thus demonstrating that these two hippocampal dependent behavioral paradigms can be used in conjunction to assess the effects of pharmacological treatments for cognitive impairment. Based on this work, researchers seeking to investigate learning and memory impairments before and after treatments will benefit from using this easily repeatable task [26]. As it is imperative to use well-developed tasks to examine individual performance within subjects before and after treatment, we expect that this study will prove to be useful to test treatments for memory impairments associated with aging and other neurological and neurodegenerative disorders.
