# Correction: A Behavioral Paradigm to Evaluate Hippocampal Performance in Aged Rodents for Pharmacological and Genetic Target Validation

**DOI:** 10.1371/annotation/50ac6b0c-19e8-43b7-833c-1aa926c51106

**Published:** 2014-01-03

**Authors:** Hilary Gerstein, Rikki Hullinger, Mary J. Lindstrom, Corinna Burger

Errors occurred during the preparation of this article for publishing. In Figure 2, label 2E is missing. In the last sentence of the results section, under the heading 'Individual Performance in the OLM Correlates to MWM Performance', the Figure referenced is incorrect. The correct Figure reference is 2E. Table 2 is incorrect. The correct version of Table 2 can be viewed here: http://plosone.org/corrections/pone.0062360.t001.cn.tiff

